# What should be included in a digital mental health intervention, based on solution-focused therapy, for young people who self-harm? A qualitative exploration of young people and clinicians’ views

**DOI:** 10.1371/journal.pdig.0001276

**Published:** 2026-03-20

**Authors:** Lauren Jerome, Katherine Adams, Victoria Bird, Dennis Ougrin

**Affiliations:** 1 Centre for Psychiatry and Mental Health, Wolfson Institute of Population Health, Queen Mary University of London, London, United Kingdom; 2 South London and Maudsley NHS Foundation Trust, London, United Kingdom; 3 Institute of Public Health and Wellbeing, University of Essex, Colchester, United Kingdom; 4 Institute of Mental Health, Ukrainian Catholic University, Lviv, Ukraine; Iran University of Medical Sciences, IRAN, ISLAMIC REPUBLIC OF

## Abstract

Digital mental health interventions (DMHIs) are an important resource for individuals who find it difficult to access in-person services, such as young people who self-harm. Most DMHIs currently are based on Cognitive-Behavioural Therapy. Solution-Focused Therapy (SFT) could provide different skills and learning but is not currently delivered digitally. Involving key stakeholders in the development of a novel DMHI is important for ensuring acceptability. The aims of this study are to explore stakeholder perceptions of a novel DMHI based in SFT, to determine what would promote or hinder engagement, and identify important content to include. We conducted focus groups and individual interviews with young people with lived experience of self-harm (*n* = 12) and clinicians experienced in SFT and working with young people who self-harm (*n* = 16). We captured a range of views through collective discussion in focus groups and more in-depth exploration in individual interviews. Data were analysed using content and framework methodologies. Content analysis enabled us to capture the direct likes, dislikes, and suggestions expressed, whereas framework analysis enabled capturing the overall perceptions of our proposed DMHI. Features identified as paramount to engagement were clear and straightforward content, personalisation to individual users, freely accessible, and assurances of confidentiality. Participants wanted content that challenged and provided novel ways of thinking. SFT was perceived as providing a different perspective to supporting mental health that participants viewed positively. Conversely, chatbots that cannot understand users and respond appropriately, or just refer people elsewhere, would discourage use. This study is the first to explore the application of SFT to a novel digital intervention, and the first to explore its acceptability with young people who self-harm. Key stakeholders felt positively about the proposed intervention, and provided specific recommendations to consider in the development of novel DMHIs.

## Author summary

There are lots of digital mental health interventions available but very few are developed and tested thoroughly. Additionally, many existing digital interventions are not used consistently by their target users. Including the target user of these interventions in the design and development phase will help them to be acceptable and engaging. Young people who self-harm may particularly benefit from digital mental health interventions since they often find it difficult to access in-person services. Solution-focused therapy is one type of therapy used with young people, and with people who self-harm, that has not yet been delivered through a digital intervention. We interviewed young people with lived experience of self-harm and clinical experts to explore what they would like a digital intervention, based on solution-focused therapy, to look like. They were positive about a digital intervention based in solution-focused therapy, and suggested it needed to be personalised, accessible, clear and straightforward, and prioritise user privacy and confidentiality. They liked the different perspective that solution-focused questions stimulated and the convenience of engaging with digital interventions. These recommendations will help us to develop an intervention that is acceptable and engaging for young people who self-harm.

## Introduction

Digital technologies to support mental health have become increasingly popular; a trend that looks set to continue. The shift towards utilising distance-based digital solutions for mental health care was accelerated by the COVID-19 pandemic [1] and the increasing demand-versus-supply gap for mental health services [2]. Announcements by the UK government in England of investment in digital mental health, as well as recommendations for how digital solutions can be utilised in the healthcare system [2], suggest digital technologies will become more and more commonplace in our health services. Digital mental health interventions (DMHIs) are appealing because they improve accessibility to support [2], are more easily scalable in comparison to non-digital interventions [3], and have some evidence of effectiveness particularly when compared to a waitlist control [4]. DMHIs are interventions addressing a mental health-related target, delivered by commonly available technologies such as mobile applications, websites, or text messages [5].

A plethora of DMHIs are already available but few have been thoroughly empirically tested. A 2020 review by Marshall et al. [6] found 293 smartphone applications available through the Apple App Store or Google Play Store that offered a therapeutic treatment for anxiety and/or depression. This does not even include applications offering singular aspects of treatment, such as mood monitoring, or applications targeting other mental health conditions. Despite the number of applications available, only 55.3% claimed to have been developed using an evidence-based theoretical framework, and only 10 had their effectiveness investigated in a published research study [6]. Although Marshall et al’s [6] review was limited to only results appearing in the App stores, and the information disclosed in the app’s description, it demonstrates a lack of published evidence to support apps that are widely available to users. The quality of these apps and whether they are delivering evidence-based support is therefore uncertain.

Moreover, it has been reported that 90% of users fail to engage with mental health applications for longer than 10 days, with some studies reporting an average retention of just 5.5 days [4]. If digital technologies are to be recommended and have impact there is a clear need to increase engagement with their intended users. One way to do this is to engage with the DMHI’s key stakeholders from the conception and design phase [7]. Understanding users’ lived experiences can uncover their requirements, challenges, and any potential barriers or facilitators to engagement that may otherwise be overlooked [7–12]. The end result are digital solutions that are more relevant to their intended user and context, integrating more easily into daily life and use, improving their overall acceptability and feasibility [7,13].

Young people who self-harm in particular may benefit from access to effective DMHIs. In the UK, 7.8% of 7–16-year-olds (based on parental report), and 32.8% of 17–24-year-olds (via self-report) reported having ever self-harmed in 2022 [14]. Young people who self-harm often experience difficulties meeting referral criteria for specialist mental health services and long waitlists to receive in-person support once they are referred [15–17]. DMHIs are one possible solution that may be helpful in providing support to these individuals because they eliminate the need for access to in-person services. DMHIs overcome additional help-seeking barriers young people who self-harm may face such as accessibility from any location, preferences to manage their problems on their own, and supporting those who may struggle to identify a trusting adult to approach for help [18].

Young people are also an ideal target group generally for DMHIs. From 11 years old over 90% of young people in the UK own their own mobile phone [19], and most young people spend more time online than older adults [20]. Additionally, young people report liking digital forms of communication when it comes to their healthcare [21], and are satisfied with DMHIs finding them acceptable and helpful [22–24].

Currently most DMHIs designed to address self-harm are based on Cognitive-Behavioural Therapy (CBT) [25]. Whilst some of these DMHIs have found positive effects post-intervention, others had to abandon testing due to problems with feasibility, or have not published findings yet. In addition, although CBT’s focus on changing thinking and behavioural patterns is helpful for many individuals [26,27], different therapeutic approaches may be valuable for others.

Solution-focused therapy (SFT) is one therapeutic approach that differs from CBT and is not currently delivered digitally. SFT focuses on exploring the individual’s ideal future and what ‘better’ looks like for them [28]. SFT is therefore flexible and considerate of individual goals and perspectives. This is important given the complexity of self-harm presentations [29], and the importance of aligning with an individual’s goals to effectively promote healthier ways of coping. The focus on their desired future and what it looks like when things go well can be empowering and motivating [30,31], compared to more traditional approaches which often focus more on altering problematic behaviours or uncovering the cause of a problem [28].

There is lots of evidence for the use of SFT with children and young people in a variety of settings [32] and some evidence for its applicability to treating individuals presenting after self-harm [31]. However, what is yet to be established is how young people who self-harm feel about engaging with SFT delivered digitally. SFT has typical questions and steps that are core to the approach [28], which in theory should be possible to deliver through a digital medium. SFT has been delivered previously through a self-directed web-based programme to college students with positive results [33], but not through a DMHI delivered to a mobile phone.

We decided to develop our DMHI to be delivered by text message since approximately 1 million children and young people in the UK do not have access to a personal smartphone or the internet at home, but usually have access to a simple mobile phone capable of sending and receiving text messages [34]. Text message-based digital interventions to support individuals who self-harm do exist, but mostly consist of simple, unidirectional interventions [25,35–37], such as reminders of supportive messages and links to support. However, two-way interaction via text message is possible that would enable a more engaging and conversational approach, like that provided by a chatbot.

Chatbots are promising since they are accessible and scalable given that they are automated, rather than requiring an individual or service to respond in real-time. Additionally, users have reported overall positive perceptions of therapeutic chatbots for mental health, liking their availability, perceived lack of judgement, ease of use, and usefulness for reflection and skills development [38]. Utilising a chatbot-style approach would eliminate the issues with waitlists and accessibility so often faced by young people who self-harm, and would allow us to develop a DMHI that could explore an individual’s goals and desires for the future, as is done typically within SFT.

We are proposing development of an automated, chatbot-style, DMHI delivered by text message, based on SFT. Studies by Kruzan et al. [39] and Hetrick et al. [40] have previously explored the preferences of young people who self-harm for a digital intervention to support their mental health. These studies focused primarily on understanding how participants managed their mental health and urges to self-injure, and how they perceived technology to help with this management. Neither seemed to explore a specific digital modality or therapeutic framework in their questions. This study therefore will be the first to explicitly explore the development of a DMHI based in SFT and delivered by text message with key stakeholders. Key stakeholders for this project are young people with lived experience of self-harm, as the target user, and mental health professionals who work with young people who self-harm, to ensure the DMHI developed is clinically relevant and has a solid therapeutic foundation.

This study aims to explore stakeholder perceptions of a chatbot-style DMHI based on SFT for young people who self-harm. This will enable us to develop a novel intervention within an evidence-based framework (SFT), and consider user requirements and facilitators in its design. This aim was achieved through focusing on the following objectives:

1. Obtain clinicians’ and young people’s views of a text message intervention based on solution-focused therapy.
2. Determine what factors will promote and hinder engagement with a DMHI delivered by text message.
3. Determine what content is important to be included within the intervention.

## Methods

The reporting of the study has been organised in accordance with the Standards for Reporting Qualitative Research (SRQR) checklist [41]. The protocol for this study was preregistered at Open Science Framework: https://osf.io/3mpwf/.

This study uses focus groups and individual interviews with young people with lived experience of self-harm, and with clinicians who have experience of working with young people who self-harm and of SFT. Focus groups were sought in the first instance as they would enable ideas and suggestions relevant to the research aims to develop through collective discussion, ultimately reflecting the perspectives of the participants [42]. Focus groups encourage discussion where opposing views can be presented and challenged, enabling a range of different opinions on a topic to be explored. This was particularly helpful for the aims of this study, where different attitudes towards digital technologies and therapeutic approaches could be explored in the discussions. Individual interviews supported the focus group discussions by providing more in-depth exploration of individual views of the topic. Individual interviews were primarily organised for participants who were unable to attend one of the arranged focus groups.

### Researcher characteristics, reflexivity, and positionality

The research team consists of individuals with backgrounds in Psychiatry (DO), clinical crisis services (KA), and Psychology (VB, LJ). The first author conducted this work as part of their doctoral research, developing a DMHI based on SFT for young people who self-harm, after coming from a research background in applied mental health clinical trials. Thus, the first author approached the research with a pragmatic, post-positivist perspective. This meant attempting to approach the research as neutrally and objectively as possible, whilst recognising and acknowledging the biases we will have as researchers, and that each individual participant will bring their own perception of reality to the discussions.

The first author has previous experience researching solution-focused approaches and so was aware that their preconceptions of these approaches could influence the discussions and results. The second author does not have this experience, which was important to bring a more balanced interpretation to the findings. Throughout the analysis the first and second author discussed their reflections on the focus groups and their coding of the data, with both perceptions incorporated into the final results.

Both coders were close to the young people’s age range for inclusion, which enabled a shared understanding. However, this also meant that they could have been more susceptible to preconceptions of young people’s preferences and behaviours that could influence interpretation of the results. The approach to analysis and use of the data dictionary helped to ensure coding was driven by the content of the discussions. Furthermore, having two coders and check-ins as coding progressed helped interpretation of the transcripts and approach to analysis be less susceptible to individual biases.

Overall, throughout the work the authors reflected on their assumptions as the study progressed, bearing in mind where the assumptions may have come from, the impact they may have on the findings, and attempting to challenge these assumptions when developing the final results. In addition, preliminary findings were shared with participants to ensure they ultimately reflected the participants’ experiences.

### Ethics statement

This study was reviewed and approved by the London - Camden & Kings Cross Research Ethics Committee. Participants provided written informed consent in advance of the focus group/interview taking place, and after discussing the study with a member of the research team and having at least 48 hours to consider participation. As per our NHS Research Ethics Committee approval, parent/guardian consent was required for any participants who were under 16 years of age at the time of consent, as well as assent from the participant themselves.

### Recruitment

We aimed to conduct four focus groups in total, two with young people and two with clinicians, to capture a range of perspectives on our proposed DMHI. We aimed to recruit 6–8 participants per focus group, which is recommended as an ideal sample size for focus groups [42]. This number would also ensure there were enough participants in each focus group for a discussion with different and opposing views to ensue, even if we encountered some drop-outs.

We took a convenience sampling approach to recruitment. Participants were recruited through adverts disseminated online through East London NHS Foundation Trust, or were individuals already in contact with the research team who had either previously engaged in, or expressed an interest in, the wider project this study is a part of. All individuals recruited through their existing engagement with the research project had been identified originally through UK-based organisations. Interested individuals who self-identified as meeting the eligibility criteria (see Table 1) contacted the research team. Although we were conscious of the representativeness of our participants, particularly in terms of gender, age, ethnicity, and professional role (for clinician participants) we did not purposively recruit based on these characteristics.

**Table 1:** Eligibility criteria.

	Age	Experience
Young People	Aged 12–25 years old	Lived experience of self-harm
Clinicians	Aged 18 years and over	Experience of working clinically with young people who self-harm AND Experience of using solution-focused techniques in clinical practice

Given that young people with lived experience of self-harm are the target audience for this novel intervention it is essential to include them in the development process to ensure their needs and requirements are sufficiently considered. Additionally, as the planned intervention will be based on SFT, it is necessary to consult clinicians with relevant experience of using this approach with young people who self-harm in order to obtain their perspectives on what works well and what is important to consider when using this approach in practice.

### Data collection

Focus groups/interviews utilised a semi-structured topic guide to steer the discussions and ensure that all relevant topics were covered, whilst allowing some flexibility in the progression of the discussion. Topic guides were developed by the authors based on existing literature around the topic, findings from earlier development phases of the wider project this study is part of, and with input from public contributors. Specifically, questions around what features participants would like in a DMHI were informed by two workshops conducted with young people prior to the study commencing, such as whether they would like it to use emojis or slang. Questions around how participants felt about typical SFT questions being delivered digitally were informed by a scoping review [31] conducted by the first author, which identified typical SFT questions and topics included when working with people at risk of suicide or self-harm.

The guide covered views of SFT, views of a chatbot-style text messaging intervention, barriers and facilitators to engagement, and content participants would like to see included in a DMHI. Topic guides were piloted with stakeholders prior to the study commencing. After the first focus group, we switched the order of questions to begin with general views of text messaging, as we felt these questions were more accessible and familiar to participants to begin discussions with.

At the start of each focus group/interview the researcher facilitating the discussion introduced the concept for this study, a chatbot-style DMHI based on SFT and delivered by text message, to provide context for the ensuing questions. The lead researcher facilitating the focus group/interview explained SFT as an overall approach as well as each type of question explored in the topic guide. An accessible graphic displaying examples of each question was shared on the screen to ensure all participants had the same understanding of it as a therapeutic approach. This graphic had been shared with patient and public contributors prior to the study taking place to validate its use.

Although questions did not focus on or require participants to share their specific personal experiences of self-harm, given the nature of the study it was possible some topics could come up which were upsetting or distressing. A thorough safeguarding protocol was developed prior to the study commencing in case any concerns for safety or wellbeing were raised. All participants were provided with a list of resources they could access for support and space was made available for any individuals who wished to take a break from the discussions (when online a separate breakout room on MS Teams was created) where the co-facilitator would also join anyone who made use of this space.

Focus groups were either held online using Microsoft Teams, or in-person at an East London NHS location. All individual interviews were held online using Microsoft Teams. Modality was chosen based on what was most pragmatic for the participants attending. All focus groups/interviews were audio-recorded using the Teams recording function, transcribed verbatim either using the Teams transcription function or an approved transcription agency, and imported into NVivo 12 for analysis. Participants were assigned a study ID which was used in the transcripts to identify the speaker. All transcripts were checked against the original recording for accuracy, at which point any references to names or locations were removed and replaced with pseudonyms. Once transcripts had been generated and checked original recordings were destroyed as per our ethical approval.

Focus groups were primarily conducted by the first author with the second author co-facilitating the online discussions. The in-person clinician focus group was co-facilitated by the fourth author. All focus groups/interviews took place between October and December 2023.

### Analysis

Transcripts were analysed using both a content and framework analysis methodology.

Content analysis [43] was used to primarily identify direct requirements expressed by the participants for content and features they wanted included in a DMHI, and what would facilitate or hinder their engagement with a DMHI. This involved coding the manifest content of the transcripts, as we were interested at this stage in the explicit preferences expressed by the participants. A data dictionary was developed which organised the coding of the transcripts into the likes, dislikes, and suggestions made by the participants. We took a conventional approach to coding which meant the codes were derived from the data and only guided by the structure of the discussions provided by the topic guide. This broadly split the results into codes related to SFT, and codes related to DMHIs delivered by text message more generally. The first and second authors both coded one focus group and one interview each using this approach and then met to discuss initial findings, ensure consistent understanding of the data dictionary, and establish how to proceed.

The coders simultaneously conducted a framework analysis to fully explore the overall views of the participants of our proposed intervention. Framework analysis classifies and organises data into a thematic framework based on themes, concepts, and categories that are identified in the data [44]. Framework analysis arranges and groups themes into higher order categories whilst retaining the raw data in an initial matrix to keep the findings in the context of the original data.

As the coders had already begun to familiarise themselves with the transcripts, for this stage they explored seemingly recurring and important themes from these transcripts by coding the latent content of the discussions. We developed an initial framework of themes in Excel following discussion between the first and second author of the themes they felt were apparent in the data. The second author proceeded to code two further transcripts and the first author coded the remainder, continuing with the content coding as well as applying our initial framework. Codes from the transcripts were copied verbatim into the initial framework. Themes were adjusted as coding continued to more closely reflect the coders observations of the data and to ensure all codes were accurately represented by the themes. Once the first and second author had finished coding and finalised the framework, the codes were summarised under each theme whilst retaining links back to the original quotes.

Codes from both the young people and clinician participants, and both the focus groups and individual interviews, were initially analysed separately and subsequently triangulated. During the discussion of codes and themes during analysis, the first and second authors explored themes present in these transcripts separately and then examined where they overlapped or differed. These similarities and differences were captured in the final results. This established the overall recommendations for a text messaging intervention based on SFT that we could use to inform development of our intervention.

Once we had produced our initial findings, the first author developed accessible documents to share these findings with the participants. This gave the participants the opportunity to suggest any amendments to the findings that would be incorporated into the final results. This was important to us to ensure the results reflected the perceptions and experiences of the participants.

## Results

Separate focus groups were held with clinician and young people participants. We conducted two focus groups with young people (*n* = 4, *n* = 5), and one focus group with clinicians (*n* = 12). Additional individual interviews were conducted with three young people participants and four clinician participants. Although we aimed to conduct two focus groups with each participant group, it was difficult to organise a second with the clinician participants due to their availability. The young people participant focus groups were originally planned to be split by age (under v. over 16 years), but due to only recruiting individuals over 16 years of age, focus groups were organised based on participant availability. The focus groups lasted around 60 minutes whereas the individual interviews lasted around 45 minutes.

### Participant characteristics

Table 2 provides details of the participants in the study.

**Table 2:** Participant socio-demographic information.

Young person		Clinician	
	N (%)		N (%)
Age			
16-19	6 (50.0)	<35	3 (18.8)
20-25	6 (50.0)	35-49	7 (43.8)
		50-65	4 (25.0)
		>65	2 (12.5)
Gender			
Female	8 (66.7)		10 (62.5)
Male	3 (25.0)		6 (37.5)
Non-binary	1 (8.3)		0 (0.0)
Ethnicity			
Asian or Asian British	6 (50.0)		3 (18.8)
Black, Black British, Caribbean or African	1 (8.3)		3 (18.8)
Mixed or multiple ethnic groups	2 (16.7)		0 (0.0)
Other ethnic group	0 (0.0)		2 (12.5)
White	3 (25.0)		8 (50.0)
First Language		Current professional role	
English	8 (66.7)	Nurse	2 (12.5)
French	1 (8.3)	Occupational Therapist	1 (6.3)
Hindi/English Bilingual	1 (8.3)	Creative Therapist	1 (6.3)
Urdu	2 (16.7)	Medical Resident	1 (6.3)
Mostly use their mobile phone for		Support worker	3 (18.8)
Browsing the internet	9 (75.0)	Systemic Psychotherapist	1 (6.3)
Gaming	5 (41.7)	Other therapist	1 (6.3)
Other apps	4 (33.3)	Psychiatrist	1 (6.3)
Music	1 (8.3)	Psychologist	1 (6.3)
Phone calls	6 (50.0)	Social Worker	4 (25.0)
Social Media (including WhatsApp)	12 (100.0)	Years worked as a clinician	
Text messaging	7 (58.3)	Range	2–50
How are calls and texts paid for		Patient population primarily worked with	
Pay as you go	1 (8.3)	CAMHS	9 (56.3)
Contract	10 (83.3)	Adults & Young adults	1 (6.3)
Only receive texts/calls or use when connected to the internet	1 (8.3)	All ages	2 (12.5)
		Mental Health	1 (6.3)
		Young people	2 (12.5)
		Children & Families	1 (6.3)
		Usual therapeutic approach	
		CBT	3 (18.8)
		DBT	2 (12.5)
		Dramatherapy	1 (6.3)
		ViG	1 (6.3)
		PBS	1 (6.3)
		IPT-A	1 (6.3)
		Art Psychotherapy	1 (6.3)
		Solution Focused	5 (31.3)
		Systemic	2 (12.5)
		Various	1 (6.3)
Total N = 12		Total N = 16	

We recruited 14 young people participants in total. Two young people withdrew from the focus groups prior to them taking place so the information is reported for the 12 who participated in a focus group (*n* = 9) or interview (*n* = 3).

All young people participants had their own smartphone, but two participants did not have consistent access to the internet at home. Participants used their mobile phones for a range of purposes, but all used it for social media (including WhatsApp). No participants identified any access needs when using their mobile phone. Only two participants reported using existing digital apps to support their health, these included Calm, HeadSpace, and the Health app provided by Apple.

We recruited 16 clinician participants in total. Clinician participants were from a range of clinical roles, predominantly social workers, support workers, nurses, and a range of different therapist roles. Most clinician participants worked predominantly with young people, and reported using a wide range of therapeutic approaches including Solution-Focused approaches, Cognitive Behavioural Therapy (CBT), Dialectical Behavioural Therapy (DBT), and Systemic Therapy, amongst others.

### Main findings

**Fig 1:**
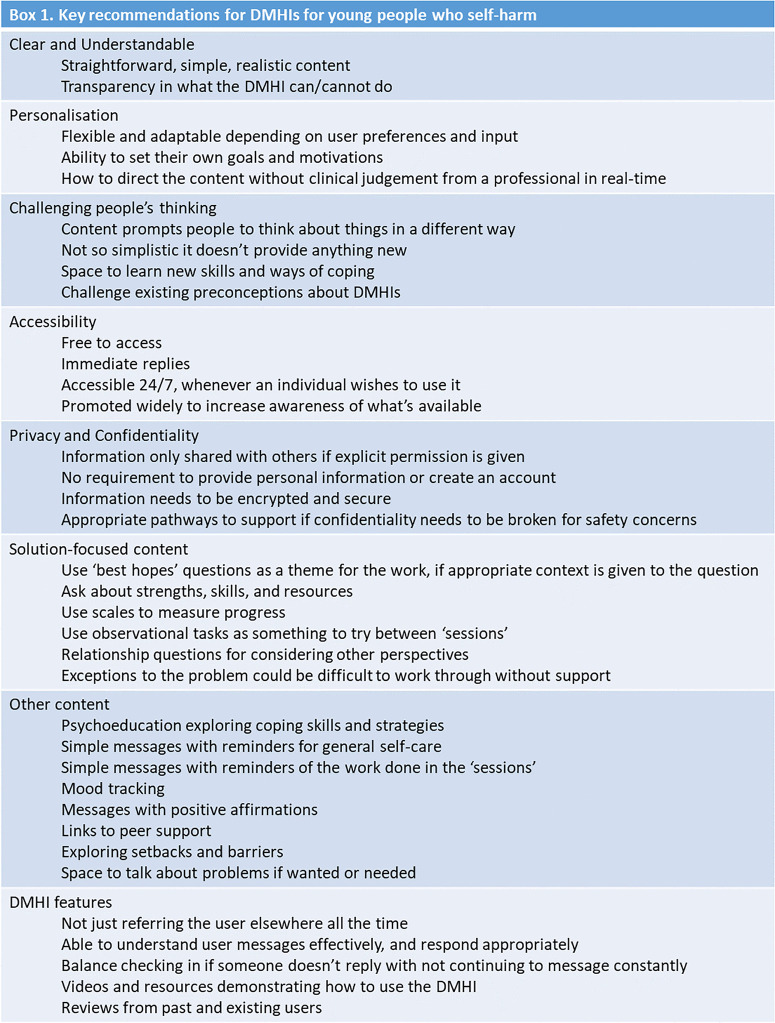
Summary of the key recommendations from our findings.

Our primary aim was to explore key stakeholder views of our proposed DMHI so their requirements can be considered throughout its design and development. A summary of the overall key recommendations is presented in Fig 1.

### Views of a text message intervention based on Solution-Focused Therapy

First we present the findings from the framework analysis which provide an overall representation of the young people and clinician participants’ views of a chatbot-style text message intervention based on SFT.

We identified five themes which encapsulated the recurring and seemingly pertinent themes in participant’s perceptions of our proposed intervention. A detailed overview of these themes is provided in S1 Appendix. All themes were present for both young people and clinicians, with slightly different emphasis and importance placed on different topics within them.

#### Clear and understandable.

This theme encompasses both the messages within the intervention themselves being clear and straightforward, as well as the intervention as a whole being clear and upfront about what it can and cannot do, and clear about who is delivering the intervention.

Clear and straightforward messages and questions that are based in reality, rather than being too abstract, will help prevent the content of the DMHI being confusing, and support the steps within the DMHI to feel realistic and achievable.

*“it should be very simple enough because a lot of people don’t like [inaudible] whereby they are being caught up in a lot of information. So I think it should be very clear and you know, not something that will end up making them panic...I feel like, if it’s not really necessary then it shouldn’t be given out. Umm, except if the person seeks for it.”* (Young Person 1, Male)

If the content is not clear and simple, this could do more harm than good by confusing users with superfluous information. It is therefore important we consider what is really key to delivering SFT in this format, and the simplest way to communicate it via text message.

*“I’d want to know if I was signing up to if I was considering it, I’d want it to be sort of say what it exactly is on the tin rather than claim to be something and then sort of me get my expectations about kind of what I’m gonna receive. And yeah, no, I think that’s that. Could can be quite damaging. So yeah, want a bit of maybe accountability and sort of what what can and it can’t do.”* (Young Person 2, Female)

Transparency in the purpose of the DMHI will help participants to decide whether it is something they want to engage with, and what it may be helpful for. This idea of accountability is particularly important. DMHI’s should be upfront about their limitations so that participants are aware of what it can and cannot be used for, and not be misled, and be clear about when help needs to be sought from elsewhere.

Young people participants also discussed how digital interventions might facilitate discussions around mental health, encouraging openness and understanding.

*“I feel like there should be a bit more knowledge and teaching and probably if this app gives it and like it becomes something that you know, like schools speak about then people are more open and like even if we go back to like if there should be a number or if it the message should be like pre named, I feel like that way if somebody wants to even see the message, they wouldn’t be as like judgemental because they’ll be like, you know, this is something that we should understand rather than be like Oh my God like, you know, have their own judgements on it.”* (Young Person 3, Female)

Many young people participants spoke of the reduced stigma associated with chatbot-style DMHIs as encouraging individual openness in discussions. This was perceived as subsequently leading to, hopefully, more openness in a wider social context to discuss mental health without judgement.

#### Personalisation.

This theme primarily covers the content within the intervention being flexible and tailored to each individual user, rather than generic messages and suggestions.

*“And it not being like again I used the word flowery but like it not being all like cookie cutter like ohh yes this will this will help you. Actually it it being quite. Deep and personal and erm thought out. Erm which seems a little bit harder for a text based thing to do, but I appreciate. But yeah, no. If I’m told to kind of have another bath or. Drink another cup of tea I will I think that will make me feel worse.”* (Young Person 2, Female)

Mental health was seen as something deeply personal and unique to each individual, where generic suggestions were deemed unhelpful and unable to teach the participants anything they did not already know. Instead, the ability for a DMHI to be personalised to each user was seen as critical for the steps within it to feel relevant for them as an individual. Additionally, it was frequently discussed that each individual will have different preferences for how they want to use a DMHI, and this will also likely change over the course of their individual journey. An effective engaging DMHI therefore needs to be able to adapt to both between- and within-individual preferences. Using SFT as the approach in a DMHI was seen to inherently support a personalised approach, as people set their own motivations and goals for the work, rather than having them imposed by a fixed agenda.

Amongst the clinicians there was also a question of whether a DMHI could sufficiently direct the conversation without clinical judgement.

*“how much your system your model is going to have, the flexibility to pick up these different responses from young people and to be able to sort of almost get almost go down different tracks according to the responses. You know how complex actually can it be?”* (Clinician 1, Male)

It will therefore be important for us to consider how to make our DMHI suitably flexible to adapt to each user whilst remaining based in a therapeutic approach.

There were also many discussions around whether an intervention delivered by text message would feel impersonal. Many participants questioned whether a DMHI could sufficiently capture the emotional side of the interaction to provide a connection that felt personal and thus support participants to feel safe to discuss their mental health. One young person participant conversely felt that the nature of our proposed DMHI, where text messages would be sent to the individual, felt more personal since the help was coming to them rather than them needing to seek it out.

#### Challenging people’s thinking.

SFT was discussed as a refreshing take on therapy, providing a new way of thinking about things that most people had not come across before.

*“it’s a good that we kind of keep giving them space to think about what they might looks like kind of hopeful way what they might want. So it’s like bring their attention towards more moving forward... because a lot of young people who kind of come to our clinic and say, I’m just really sick and tired of saying the same thing over over again to different like clinicians, which is actually really true”* (Clinician 2, Female)

Typical SFT questions like the ‘best hopes’ (what are your best hopes from us meeting today?) or ‘miracle’ questions (imagine you wake up tomorrow and the problem that brought you here has gone, what is the first thing you would notice?) were seen as helpful for focusing on what the individual wants for themselves in the future and on positives, rather than feeling stuck in the past and focusing on negatives. Other typical SFT questions like ‘relationship’ questions (what would someone close to you notice is different about you?) were also seen as helpful for thinking about things from a different perspective, that can be difficult when you are caught up in your current situation. Clinician participants discussed how SFT can promote power and agency amongst the user, who is encouraged to reflect on their individual hopes and desires, which are then used as the theme for the work.

However, amongst some of the young people there was discussion of whether as an approach it is too simple, and does not provide anything new.

*“But for me, it’s a bit of common sense, like focus on the positives basically.”* (Young Person 4, Female)

This suggests we need to think carefully about how we might be able to convey the power of the questions and approach used typically in SFT in an autonomous DMHI without it feeling too simplistic.

There was also discussion of digital tools being a space for learning new skills and ways of coping, but also some uncertainty of whether they would have an impact.

*“I think probably the main thing is probably like, well, couple of things would be like one is sort of learning about different maybe strategies they can use. To like sort of like an action plan”* (Young Person 5, Male)

Young people participants talked about how receiving new information from a trusted source was important, as opposed to finding it on the internet where the source is unknown. Although, other young people participants highlighted the value of seeing suggestions from peers where there was perceived to be more mutual understanding.

Additionally, the young people participants recognised that existing preconceptions of whether they thought a DMHI would be helpful would impact whether they would even try it out in the first place. This highlights the need to think carefully about how to promote DMHIs to challenge these preconceptions and encourage the target user to give them a go.

#### Accessibility.

This theme was predominantly present in the discussions with young people participants. Overall, digital interventions are perceived as a way to access support quickly, at a time that is convenient, as long as people are aware of what is available to them. However, they need to remain free to access, and alternatives for individuals who do not have compatible technology need to be considered.

*“the biggest pulling factor would just be the the timeliness of it. So just being able to access something there and then that that would definitely be a massive pull factor because there’s it just seems like. So it seems like such hard work being able to get any sort of help and such a long winded process. So yeah, just that would be the main thing. Just knowing that no matter where I am, what time it is that I can, I can use that and it can be like a reasonably fast response as well.”* (Young Person 6, Female)

The main perceived benefit of DMHIs is their ability to be used flexibly, at the moment that an individual needs them. Clinician participants also felt that anything that improved young people’s access to services was a positive. This supports our decision to pursue an automated chatbot-style DMHI which does not require a person replying in real-time, as it can be available to respond immediately 24/7.

Several young people participants also spoke about needing to be aware of what DMHIs were available to them, and the importance of knowing their options for support in order to be able to decide what would help them best. This again highlights the necessity of considering of how DMHIs are promoted, so that those who may benefit are aware of them.

#### Privacy and confidentiality.

The final theme was also mostly present in the discussions with young people participants. This theme encompassed who should or should not be able to see your data, how secure the digital system is, as well as the point that being anonymous can help people feel more comfortable opening up to talk about difficulties they are facing.

*“Yeah, I think it kind of takes away that, like, um, reliance on the person you’re talking to if it’s like anonymous... I feel like you’d kind of share more because they don’t like, know you and stuff.”* (Young Person 7, Female)

Young people wanted to be reassured their information would only be shared with others if they gave their explicit permission, and were hesitant to engage with a DMHI if it required them to provide personal details or set up an account. Outside of the DMHI, there was also discussion around family or peers seeing content on their phone, and how this could be kept confidential. The young people in this study were well informed of data protection principles and wanted to ensure any digital interventions they used were encrypted and kept their information secure.

Clinician participants also spoke about confidentiality in the context of needing to know if someone in their care was using a digital intervention, and ensuring appropriate pathways are in place for when someone is at risk and confidentiality might need to be broken.

*“Yeah, so because if they say, “I’m going to kill myself now” on the chat, what happens there?”* (Clinician 3, Female)

When and how to escalate support, especially in situations of risk or safety concerns, requires careful consideration and will make working with clinical services to implement DMHIs vital. It also highlights a need to balance an individual’s right to privacy and confidentiality whilst ensuring safety and appropriate support is prioritised.

### What content should be included?

Next we present the findings from the content analysis. The content analysis primarily identified the direct likes, dislikes, and suggestions proposed by the participants to provide us with what to consider including or omitting from our proposed DMHI. A full list of results from this stage of analysis is available in S2 Appendix. We highlight some of the key findings, organised by codes related to the type of therapy, and those related to DMHIs more generally.

#### Solution-focused therapy.

Participants liked using the ‘best hopes’ question as a theme for the work, but felt going straight into this question in a DMHI could be difficult if not much context was given. They preferred this over the ‘miracle question’ as a method for getting users to think about the future that they want.

*“I like the idea that it makes you more centred towards what you want to have at the end of the therapy. And I think you can focus a lot on like the progress you make out of it and starting with that question”* (Young Person 8, Female)

Asking about strengths and skills was seen as important, and individuals liked the use of scales to measure where they are and their progress. Participants liked the use of more passive, observational tasks as something to try in-between ‘sessions’ with the DMHI, and liked the use of relationship questions to think about things from other perspectives. Asking about times when the problem was absent or less severe was generally viewed as quite a powerful question. However, it was felt it could be quite difficult or upsetting to discuss, which may be problematic when delivered digitally if there is not a person there to provide real empathetic support.

*“I think it is good, but I feel like at the same time umm, I don’t know. Like, I feel like sometimes it might bring up bad memories. Like, I feel like it might make someone feel a bit upset. Like you know, I wish I was like that. Or I wish times were like that, and I feel like sometimes that just throws someone into like a deep end or something”* (Young Person 3, Female)

There were a number of therapeutic components discussed that are not strictly SFT but were deemed important by the participants. Psychoeducation on coping skills and helping people identify strategies that work for them was seen as important. Crucially these needed to be developed and explored with the user rather than simply telling people what to do or not do. Additionally, simple text messages reminding them of general self-care and things they had specifically addressed in the session, mood tracking, messages with positive affirmations, linking with peer support, and preparing for setbacks and barriers were also mentioned as content they would like to see in a DMHI. Participants also felt it was important to give space to share their feelings and talk about problems if that was what they wanted to do, rather than always going straight into solutions.

#### Digital interventions more generally.

Both young person and clinician participants highlighted the need for appropriate pathways and additional support to be in place for individuals that need more help than the DMHI could provide. However, this was important to balance with being referred elsewhere all the time, which participants felt would invalidate the use of the intervention itself.

*“I feel like sometimes links can help, but I feel like with me like I know I get, I do get annoyed when I’m always being given links...I’m trying to get help from this place, but they’re just sending me elsewhere...you might think I’m on this text messaging system and rather than it helping me itself, it’s just sending me to other external links”* (Young Person 3, Female)

There was lots of discussion around the need for a digital intervention to pick up on cues and answers in order to respond appropriately. Numerous participants reflected on existing digital tools which fail to do this as frustrating and annoying. Additionally, participants did not want a digital intervention to keep sending them messages if they were not replying, although this was balanced with a need to check in and make sure someone is okay if they have not responded in a while.

*“In general...I find it really frustrating because they often don’t really answer that. What I ask....and it it can be more. Bring some more frustration”* (Clinician 2, Female)

A number of ways to personalise the intervention, beyond the intervention responding appropriately, were proposed. Participants wanted to be able to set how formal or informal the language of the intervention was, with some preferring it to sound more professional and others preferring it to sound like a friend. Participants also wanted to be able to set when they would receive messages, and wanted to be able to react to messages they particularly liked or did not like, to see more or less of that content in future.

Finally, participants discussed the need for clarity in exactly how the digital intervention would work and what they could or could not use it for. Videos and resources demonstrating how to use the different aspects of it were deemed helpful, as well as trialling messages or being given demonstrations of the intervention. Reviews from existing or past users endorsing the intervention were seen as crucial to encouraging others to try an intervention for themselves.

*“And also I will also like to see a [inaudible] like a past review from people who have actually tried the service and say it’s beneficial and this will also give me the, you know confidence about wanting to take part in it”* (Young Person 1, Male)

## Discussion

This study explored what young people with lived experience of self-harm, and mental health professionals, want a DMHI based on SFT to look like. Overall, the perception of a chatbot-style DMHI, delivered by text message, based on SFT, was positive. SFT delivered through a digital medium was seen as a different approach that could prompt the user to think about things in a new way. Considering the requirements and preferences highlighted in our findings when we develop our DMHI will help our final DMHI to be acceptable, engaging, and effective.

Personalisation was one of the most prominent themes in the discussions we had with participants in this study. This included both wanting the content of the messages within the intervention to be personalised, as well as discussions of whether digital interventions could be personalised enough to be engaging and impactful. The Affect-Integration-Motivation and Attention-Context-Translation (AIM-ACT) framework conceptualises engagement with digital stimuli as a multi-stage process, and proposes engagement is most likely to translate to actual behaviour when outcomes are internalised and valued by one’s self [45]. This suggests goals personalised to a user that align with their values will have the best chance of being achieved. Given the complex and unique nature of how young people understand their self-harm thoughts and behaviours [39], personalisation of a DMHI may be particularly important in this context in order for it to be relevant to their individual needs and goals. SFT centres primarily around whatever the client wishes to discuss, helping it to align with their values. It is well established that accommodating client preferences within traditional psychotherapies has a positive impact on treatment adherence and outcomes [46]. It is therefore important that the same accommodation needs to be made in DMHIs.

However, the evidence for the impact of such personalisation within DMHIs is lacking, which may in part be due to discrepancies in how the term ‘personalisation’ is used and understood in DMHI research [47]. Furthermore, most existing digital interventions use rule-based or user choice methods of personalisation, making adaptations quite simple (i.e., different content if a user is above or below a certain symptom score), rather than employing more complex models that could additionally account for a wider variety of user characteristics and preferences (for example including sensor data like GPS location or mobile phone usage like the number of texts or calls made) [47]. Using more complex models requires more complicated programming and data input, but the result would be an intervention with an illusion of better memory and rapport with less effort required by the user [48]. Although more effort is required by the developer, our participants felt tailored and customised content would make a novel DMHI more appealing. This fits with existing research suggesting that generic content does not tell users anything new and is not wanted, instead customised content is key [39,40]. In our proposed DMHI, this could include using both algorithms and scale scores to determine whether users are struggling at that moment, based on their responses to the chatbot, and thus direct them to messages exploring coping rather than moving forwards. Clarity in future research of how exactly DMHIs are personalised will help to evaluate which aspects of personalisation are important.

Privacy and confidentiality was another topic that featured consistently in the discussions we had in this study, particularly with the young people participants. Privacy and security are considered by the NHS Confederation as one of the biggest challenges to successful integration of digital technologies into mental healthcare [2]. Although data protection regulations and policies exist, there needs to be an approach to ensure digital interventions adhere to these standards [2,49]. In recent years there has been an increased focus on developing regulatory guidance to match the development of digital solutions, including project funding such as that granted by the Wellcome Trust to produce guidance regulating digital mental health tools specifically [50]. Including key stakeholders in this work will ensure the resulting regulations reflect the needs and priorities of the end users. What is apparent from our findings is that young people have clear expectations for how their information is kept private when using digital technologies. This highlights a need for DMHIs to prioritise privacy and confidentiality for their users, whilst including appropriate pathways to break confidentiality in situations where concerns for safety and risk take precedence.

Clinician participants in this study highlighted a need for clarity around how and when users would be escalated for additional support, and what happens if users disclose something to the intervention which raises a concern for their safety. In order for digital interventions to be successfully adopted and sustainable, the health/care systems and wider context they will be delivered in are important to consider from the design phase [51], as well as the individual user. Careful consideration of how to balance user preferences (i.e., users may not want to provide personal details) so benefits such as encouraged disclosure to the intervention are retained whilst prioritising safety will be key to engagement and adoption. In the development of our intervention, the safety issues raised (such as what happens if someone says they are going to harm themselves) will inform the approach to risk escalation integrated into the intervention that can then be tested with young people for acceptability in its testing phase. This could include an automatic triage of messages flagged for safety concerns to a clinical team when a user’s identity is known, or responses from the chatbot being triggered which include helplines to contact in a crisis, and exploration of a person the user can contact if they feel unsafe.

There are some commonalities in our findings with the studies conducted by Kruzan et al. [39] and Hetrick et al. [40]. Kruzan et al. [39] found participants used technology to learn about self-injury and alternative ways to cope, which is similar to our finding whereby participants would like the option to receive educational content on coping. Both Kruzan et al. [39] and Hetrick et al. [40] found that participants valued peer support for feeling they are not alone, but recognised this could become negative if not appropriately moderated. We also found participants wanted a space to talk to peers going through the same difficulties. Kruzan et al. [39] also found a range of activities that were specific to the user were deemed most helpful and engaging, which needed to reflect changing needs and goals over time. This is similar to our finding that participants wanted personalised content, and valued the use of solution-focused questions like the ‘best hopes’ question to make their unique goals and needs the theme for the work in the intervention. Both Kruzan et al. [39] and Hetrick et al. [40] findings share similarities with those reported in our study. We add to this existing work by specifically exploring the delivery of SFT in a digital format, and report direct recommendations of the desired content for a DMHI. Specifically one delivered as a chatbot via text message.

To our knowledge this is the first study to provide evidence for the perspective of young people who self-harm on SFT as an approach to support their mental health. Previous work has explored the perceptions of adults [30] and young people with learning difficulties [52] of SFT, after they had engaged in SFT sessions. In those studies participants liked being supported to define their aims, the attitude towards change and the future, and focusing on strengths, resources, and moving forward. This is similar to the findings of our study. Participants liked the focus on the future and strengths, and the use of solution-focused questions felt like a new and different approach to what they were used to, providing an opportunity to change their perspective and challenge their thinking. Some questions, such as exploring exceptions to the problem, were considered challenging; highlighting careful consideration of techniques that might require more support to explore. These findings add to the evidence-base for the use of SFT with young people who self-harm.

However, some participants in our study also highlighted that SFT could feel a little simplistic. Some participants stated that they knew focusing on positives for example was a good step to take, but either struggled to see the benefit of this approach or were unlikely to find these kinds of questions engaging. A challenge in developing our proposed DMHI will be to convey the therapeutic power of the approach whilst being simple and straightforward, and keeping users engaged in a self-directed autonomous delivery of the approach. Referring again to the AIM-ACT framework [45], digital stimuli need to balance complexity and simplicity. A positive affective response to stimuli will increase the likelihood of motivation to carry out the end behaviour. Nahum-Shani et al. [45] propose this is most likely if the intervention is not either frustrating or results in boredom. This suggests we need to think carefully about how questions are framed and concepts explored so that the approach feels novel and not boring. For example, in SFT typically the therapist will ask the client ‘and what else?’ to uncover additional detail of what their ideal future and better looks like for them. In a DMHI we will need to balance including these questions to probe for further detail, with moving on or sufficient rephrasing if the user cannot think of something so the conversation does not feel repetitive and frustrating.

Additionally, other types of questions and activities that are not typically used in SFT were still seen as valuable by our participants (e.g., education on coping, mood tracking, peer support). This raises a question of whether developing a DMHI strictly within one therapeutic approach is most effective, or whether a more integrative approach is beneficial. It will be important when examining effectiveness to try to determine which aspects of the DMHI are beneficial, and whether the SFT content alone is sufficient to result in positive outcomes. Studies like O’Sullivan et al’s [53] have used methods such as causal analyses to begin to try to determine which aspects of DMHI’s influence engagement and outcomes. Similar methods could be employed at the evaluation stage of other DMHIs.

Despite the clear importance of involving end users in DMHI design and development to promote acceptance and engagement, how we measure and test these constructs is important. A review by Bear et al. [13] of mental health apps for young people, focusing on assessing implementation, consistently found that engagement decreased over time. Whilst this may appear concerning, inconsistency in how engagement is conceptualised makes it difficult to determine whether this disengagement is in fact a problem [13]. Choosing which metrics to measure acceptance and engagement by is important, and in fact lack of usage may actually represent a positive outcome [3]. For example, a mobile application containing a safety plan for individuals at risk of suicide may not be used because the individual does not need to use it, not because they dislike the app itself. When evaluating DMHIs it needs to be decided apriori what good engagement looks like, or whether measures of improvement are in fact more appropriate to determine the DMHIs success [54]. Measuring both usage of the DMHI as well as user wellbeing or symptom improvement could help to determine whether users simply do not feel a need to engage with the DMHI, or whether they have not engaged because they do not perceive it as helpful. Furthermore, obtaining feedback on the DMHI from users will be most helpful to establish its acceptability, and in what scenarios users may or may not engage with it.

### Strengths and limitations

This study has several strengths. Primarily it is, to our knowledge, the first study to explore the perspectives of young people who self-harm of SFT and its application to an autonomous chatbot-style DMHI delivered by text message. Most existing work provides broad recommendations for the development of DMHI’s for young people who self-harm. We aimed to provide specific direct suggestions that can also be utilised by other researchers working in this field. Additionally we triangulated our findings using both data from young people and clinicians, and from focus groups and individual interviews, to ensure our results captured perspectives from all our included stakeholders. We also invited the participants to comment on our initial findings to ensure our results reflected their perceptions and experiences. This was important to keep our stakeholders at the centre of our work. Finally, we recruited participants from a range of backgrounds and experiences, which brought a rich diversity to our discussions.

However, a primary limitation of the study was in its recruitment approach. Recruitment was based on convenience, occurred through online advertisements, and most discussions were held online. We would expect our participants therefore to have some familiarity and acceptance of digital technologies. This introduces a potential bias in our findings whereby participants are more likely to view digital interventions and delivery of therapies more favourably. Additionally, all participants in our study had their own smartphone and used their phone for social media, including WhatsApp. This could also suggest our participants are familiar with digital technologies, using mobile phones, and messaging. This may have also influenced our results compared to if we had included young people who did not regularly use smartphones or social media, who may have different preferences for accessing support remotely. Future studies should seek to recruit participants and hold focus groups offline to encourage young people who use technology less to participate, for example making use of community organisations and spaces.

Our findings are also limited to a UK-context, and to young people aged over 16 years. The development of DHMIs in other geographical locations and to individuals younger than 16 years may find other preferences and requirements are important. Finally, two young people participants dropped out of participating in the focus groups. This suggests our participants may represent individuals at a specific point in their recovery journey and we may need to consider this when developing our intervention; at what point and for whom will this be appropriate.

## Conclusions

Young people with lived experience of self-harm, and mental health professionals, were positive about a novel chatbot-style DMHI, delivered by text message, based on SFT, to support the mental health of young people who self-harm. Our participants’ positive perceptions of SFT and suggestions for its delivery adds to the evidence-base for its use with young people who self-harm. Participants in this study provided us with numerous recommendations that can be actioned in the development of DMHIs to improve the likelihood of acceptability and engagement. In particular, careful consideration of how to make our DMHI suitably personalised to each user, how to deliver SFT simply but with enough complexity to remain engaging, and how to determine the effectiveness of our DMHI and its components is needed.

## Supporting information

S1 Appendix Overview of the themes identified in the framework analysis of the focus group and interview transcripts. (DOCX)

S2 Appendix Findings from the content analysis of the focus group and interview transcripts. (DOCX)
