# Supplementary material for: What should be included in a digital mental health intervention, based on solution-focused therapy, for young people who self-harm? A qualitative exploration of young people and clinicians’ views
Source: PLOS Digit Health. 2026 Mar 20;5(3):e0001276. doi: 10.1371/journal.pdig.0001276 (PMC13004387; doi:10.1371/journal.pdig.0001276)
Supplement: S1 Appendix — (DOCX) [file pdig.0001276.s001.docx]

S1 Appendix. Overview of the themes identified in the framework analysis of the focus group and interview transcripts.

| Theme | Young People | Clinicians |
| --- | --- | --- |
| 1) Clear and understandable | Want someone to go through how and why to use the tool | User needs to understand why they’re in therapy and using the tool |
|  | Some questions wording is too complicated (miracle question)/others are more realistic and help with clear communication | Question wording needs to be clear, realistic and remain in things that are possible, and not too abstract |
|  | Content should be clear, simple and only included if key | Should establish clear goals from the user for using the tool |
|  | Want to know what the therapy can offer you, be upfront about what they can and can’t do | Need to know strengths and weaknesses of the tool, should be upfront about being a robot, and direct elsewhere when human intervention is needed |
|  | Want to see reviews from other users |  |
|  | Digital therapies facilitate openness discussing mental health |  |
|  | Want to know where the messages come from i.e. name on the sender, which organisation do they come from |  |
| 2) Personalisation | Should be able to set the tone and language of the messages to fit individual preferences | Should be able to set the tone of the conversation |
|  | Therapy delivered by text doesn’t feel as personal/impersonal | Texting feels impersonal |
|  | Approach focuses on what the individual wants and their progress | Young people should be the centre of their therapy conversations |
|  | Questions should be tailored to consider the individual’s situation i.e. relationships referred to in others perspective questions, also suggestions based on what they have said | How well the approach works will depend on the individual’s situation and needs, and if chatbots can be flexible, respond appropriately and remember what individuals say |
|  | People should be given options so they can pick what they want to do, and which messages they want to receive | People should be given options for therapy in person or by text, and what tasks etc to do within it, to choose what suits them |
|  | Extra content e.g. educational material should only be sent if requested |  |
|  | Should be able to choose how often messages are sent, will depend on individual need |  |
|  |  | Need clinical judgement for what approach/questions are appropriate for someone |
|  |  | Texting will miss contextual factors you get in in-person therapy |
| 3) Challenging People’s Thinking | Questions in SFT get people to think about things in a different/new way, but some are difficult to answer | Questions in SFT can be good for getting people to think about things differently and giving them agency, but can sometimes be hard to answer |
|  | People want to learn new coping strategies | Education on coping is important |
|  | People are sceptical of how much digital tools can help | Need to have goals related to decreasing self-harm/need to know the outcome the person wants for therapy to be helpful |
|  | Need to understand the problem before moving on to solutions/focusing on the problem makes people feel stuck | Need clinical judgement of when time needs to be given to the problem |
|  | Some people feel SFT is too simple and doesn’t tell them anything they don’t already know |  |
|  | Having lots of ways to use digital tools will increase engagement |  |
|  | Currently, some people only use non-professional sources of support |  |
| 4) Accessibility | Digital support is a way to get therapy quickly | Feel positive about anything that will improve accessibility to therapy |
|  | Digital support is convenient and can be accessed flexibly | Young people like access to things when it is convenient for them |
|  | Need awareness of digital tools available in order to access support |  |
|  | Need to consider people who don’t have a phone |  |
|  | Paywalls stop people accessing help |  |
| 5) Privacy and Confidentiality | Professionals should/shouldn’t be involved in your use of digital tools | Need access to the work the young person does in the tool for it to be helpful in therapy |
|  | People feel more comfortable opening up anonymously |  |
|  | Concerns around privacy from family or anyone who might see your phone |  |
|  | What details and data will the tool keep, are messages encrypted |  |
|  |  | Need to make sure appropriate pathways are in place for people in crisis or at risk |
