# Supplementary material for: What should be included in a digital mental health intervention, based on solution-focused therapy, for young people who self-harm? A qualitative exploration of young people and clinicians’ views
Source: PLOS Digit Health. 2026 Mar 20;5(3):e0001276. doi: 10.1371/journal.pdig.0001276 (PMC13004387; doi:10.1371/journal.pdig.0001276)
Supplement: S2 Appendix — (DOCX) [file pdig.0001276.s002.docx]

S2 Appendix. Findings from the content analysis of the focus group and interview transcripts.

| Wants | Mixed views | Don’t wants |
| --- | --- | --- |
| Space to share feelings and talk about difficulties | Asking about times the problem was absent – maybe only ask if someone decides to disclose a difficult time they are currently having | - Ask directly about problems repeatedly - To go straight into solutions if someone just wants to talk about how they’re feeling |
| Focus on the individual’s best hopes for themselves and what they want to get out of using it | Miracle question | Going straight into beginning with best hopes for using the tool is difficult |
| Able to set the tone of the conversation | How informal/formal the language should be – can the person set the tone | Really formal/informal language, lots of emojis or slang |
| Assurances of privacy, confidentiality, data security, anonymity | Whether or not it should come from a prenamed number i.e. NHS | To have to make an account or provide personal details |
| Appropriate pathways and support for individuals needing more help |  | Be referred elsewhere all the time |
| Psychoeducation on coping and alternatives – provide tips and links to further information if requested, help people come up with ways to cope that work for them, review and refine strategies |  | Telling people what they should/shouldn’t do |
| To work alongside any contact with existing services |  | To share information with clinicians/services without their express permission |
| Needs to respond appropriately, pick up on cues and remember the person’s individual context and previous answers |  | Prescripted generic messages and answers |
| Accessible whenever people want to use it - Use flexibly depending on when the person feels they need it |  | Would muddle things if used at the same time as face-to-face therapy |
| Choice to not answer some questions if they don’t want to |  | To keep messaging if someone isn’t replying – check in but don’t keep sending texts |
| Focusing on positives, the future, wants, using hopeful language |  |  |
| Asking about strengths and skills |  |  |
| Reminders – not too many, of day-to-day things (e.g. eating, check in on yourself), therapuetic things they said help (e.g. few deep breaths), and of things they’d been working on in the session |  |  |
| Scaling questions |  |  |
| Noticing tasks/tasks they come up with |  |  |
| Others perspective questions – need to check person’s relationships first |  |  |
| Track progress and review past sessions |  |  |
| Daily prompts to track mood |  |  |
| Sending positive affirmations |  |  |
| Begin by checking in with how someone is doing, if they respond can start a conversation |  |  |
| Linking in with peer support |  |  |
| Provide examples of what others have said if stuck |  |  |
| Reviews from users of the tool |  |  |
| Ability to use reactions/respond to message you like or don’t like |  |  |
| Think about small steps and focus on small changes |  |  |
| Prepare for setbacks and barriers |  |  |
| Able to set how often they receive messages and schedule the next one |  |  |
| Upfront about it being a chatbot and what it can/can’t do |  |  |
| Clarity in exactly how it would work i.e. how many messages, how often, what times etc |  |  |
| Immediate replies |  |  |
| Use your name |  |  |
| Have someone go through how to use the tool, as well as videos/resources to show why you use it and how it works |  |  |
| Give personalised suggestions depending on how they said they’re feeling |  |  |
| Ability to trial the messages before joining/ Clinician training/demo of the tool |  |  |
| Advertised widely |  |  |
|  |  | To pay for content |
